# Oral Manifestations of Systemic Lupus Erythematosus Patients in Qatar: A Pilot Study

**DOI:** 10.1155/2018/6052326

**Published:** 2018-04-10

**Authors:** Mohammed Hammoudeh, Ahmed Al-Momani, Husam Sarakbi, Prem Chandra, Samer Hammoudeh

**Affiliations:** ^1^Department of Medicine, Hamad Medical Corporation, P.O. Box 3050, Doha, Qatar; ^2^Department of Dentistry, Hamad Medical Corporation, P.O. Box 3050, Doha, Qatar; ^3^Medical Research Center, Hamad Medical Corporation, P.O. Box 3050, Doha, Qatar; ^4^Research Department, Weill Cornell Medicine-Qatar, P.O. Box 24144, Doha, Qatar

## Abstract

**Objective:**

The purpose of this pilot study was to assess the prevalence of oral manifestations among systemic lupus erythematosus (SLE) patients in Qatar, in order to warrant future studies that would investigate each one of these manifestations with detail and further scrutiny.

**Methods:**

Study procedures took place between November 2014 and April 2016. All patients visiting the outpatient rheumatology clinics at Hamad General Hospital, Doha, Qatar, were asked to join. The American College of Rheumatology (ACR) 1997 criteria of SLE were used. The patients were examined initially by a rheumatologist and were later scheduled for an appointment with a dentist at the same institution. A total of 77 patients were recruited for the study.

**Results:**

Prevalence rates for the different oral manifestations ranged from 2.4% for soft palate ulcers, cheilitis, and oral candida to 88.1% for the presence of cavitation. Gingivitis, periodontal disease, cavities, and missing teeth were observed in more than 50% of the sample. The prevalence of periodontal disease and missing teeth was higher among those with an SLE duration > 8 years. On the contrary, the prevalence of gingivitis and cavities was higher among those with an SLE duration ≤ 8 years.

**Conclusion:**

This study found high rates of gingivitis, periodontal disease, cavities, and missing teeth among SLE patients in Qatar. It is recommended that healthcare providers of such patients monitor the presence of any oral manifestations in order to arrange for early treatment and prevention efforts. Future prospective longitudinal studies with adequate sample size and power are needed in order to ascertain any causation factors or common etiology pathways.

## 1. Background

Systemic lupus erythematosus (SLE) is a chronic autoimmune inflammatory disease which involves the connective tissue [1–3]. Several organs are also involved such as the brain, lungs, kidneys, heart, blood vessels, muscles, and skin [4]. SLE is more common among women [5–7], with a common onset between 15 and 40 years of age [4]. Researchers reported a prevalence rate of 30–50 cases per every 100,000, while incidence rates ranged from 0.9 to 3.1% among every 100,000 per year, among Asian populations [8]. In Saudi Arabia, a prevalence rate of 19.28 per 100,000 has been reported [9]. Globally, a prevalence rate of 12–50 cases per every 100,000 has been reported [10], while an incidence rate ranging from 0.3 in Ukraine to 11 in Australia cases per every 100,000 has been reported [11]. Common symptoms of SLE include fever, weight loss, glomerulonephritis, alopecia, rash, and vesiculobullous lesions [12]. Other manifestations such as arthralgia and arthritis are frequently seen, with migratory arthritis affecting about 75% of patients [4].

Oral manifestations of SLE are frequently encountered [13] and may include oral ulceration, honeycomb plaque, raised keratotic plaque, nonspecific erythema, purpura, petechiae, and cheilitis [14]. Research shows that 25% of SLE patients have oral mucous membrane and lip involvement with possible petechiae. Both xerostomia and hyposalivation predispose patients with SLE to dental caries and recurrent noninfectious pharyngitis and oral ulcerations. Oral candidiasis and infections are also common due to the usage of corticosteroid which is used in the treatment of SLE [4]. The most common oral manifestation reported to be present among SLE patients is oral ulcerations [3], with a prevalence rate ranging between 7 and 41%, which is observed to be more severe as the disease [15]. Others reported a prevalence rate of 8–45% for oral lesions among SLE [16].

A study conducted in Venezuela on 90 systemic and cutaneous lupus erythematosus patients reported that 11% had oral lesions including oral ulcerations, erythema, and white plaque [17]. A study conducted in Saudi Arabia on 46 SLE patients reported mucocutaneous involvement including oral ulcers in 72% and discoid lupus in 13% of the sample [18].

Among the oral manifestations is periodontal disease (PD), which is an infection of the tissue supporting and surrounding tooth structure [19]. Sales et al. showed that a relationship does exist between SLE activity and periodontal status, along with a relationship between the latter and levels of CRP in serum [20]. In an earlier study, Rhodus and Johnson showed a high prevalence of oral lesions among SLE patients, including angular cheilitis, ulcers, mucositis, and glossitis. A high prevalence of oral complaints such as dysphagia, dysgeusia, and glossodynia was also present [15]. A recent systematic review reported a significant association between periodontitis and SLE [13].

Fabbri et al. showed that treatment of periodontal disease among SLE patients on immunosuppressive therapy is beneficial in controlling disease activity [21]. A more recent study as well showed that treatment of periodontal disease aids in reducing the symptoms of SLE [22].

On the contrary, Mutlu et al. showed no significant difference in periodontal probing depths between SLE patients and healthy controls, yet concluding the absence of any evidence that points toward higher predisposition of SLE patients to periodontal involvement [23]. A recent review reported conflicting results as well [24]. Al-Mutairi reported no significant difference in periodontal findings between 25 SLE patients and 50 healthy controls. Among those with SLE, no correlation was found between SLE biomarkers and their periodontal findings [25].

The purpose of this study was to assess the prevalence of oral manifestations among systemic lupus erythematosus patients in Qatar in order to warrant future studies that would investigate each one of these manifestations with detail and further scrutiny.

## 2. Materials and Methods

The study took place between November 2014 and April 2016. All patients visiting the outpatient rheumatology clinics at Hamad General Hospital, Doha, Qatar, were asked to participate. A waiver of consent form was obtained from the IRB at the same institution, which also provided IRB approval for the study (14301/14). The inclusion criteria for the study included subjects (a) meeting the American College of Rheumatology (ACR) 1997 criteria of SLE, (b) willing to participate in the study, and (c) able to tolerate study oral and dental examination procedures. The exclusion criteria excluded subjects who (a) did not meet the 1997 ACR criteria of SLE, (b) were unwilling to participate in the study, and (c) were unable to tolerate study oral and dental examination procedures. The patients were examined initially by a rheumatologist and were later scheduled for an appointment with the same dentist at the same institution, for an oral and dental examination.

The rheumatologist consented subjects checked that they meet the ACR 1997 criteria for SLE and recorded the following information: date of diagnosis, the presence of any comorbid conditions, the status of disease activity on the date of the examination based on clinical and serological data, medications, demographic, and education level. A clinical oral and calibrated periodontal exam was done by the same dentist. The dentist was requested to document the following oral manifestations on a data collection sheet that was designed for the study: soft and hard palate ulcers, ecchymosis, petechiae, lupus mucosae oris, cheilitis, candida, discoid, herpes simplex, gingivitis, periodontal disease, cavities, and missing teeth. No biopsy was taken. A total of 77 patients were recruited for the study. Only 42 patients appeared for their dental appointments. The findings in those 42 patients are presented and discussed in this publication.

### 2.1. Statistical Analysis

Anonymous data were collected and entered into a standard electronic database designed in view of study design and objectives. Descriptive statistics were used to summarize all demographic, clinical, and other characteristics of the participants. The primary outcome variable was to assess and estimate the prevalence of oral manifestations among systemic lupus erythematosus patients among the population in Qatar, and this was estimated and tested using appropriate *Z* test and the corresponding 95% CI was computed to measure the precision of the prevalence estimate. The prevalence of different oral manifestations (%) was calculated by dividing the total number of oral manifestation cases (prevalent cases) by the total number of participants included. Associations between two or more qualitative variables (gender, different oral manifestations with dichotomous categories of disease duration, etc.) were assessed using chi-square (*χ*^2^) test, Fisher Exact test, and/or Yates corrected chi-square as appropriate. Quantitative variables (age, SLE duration, etc.) means between the two independent groups (SLE active and inactive groups) were analyzed using unpaired “*t*” test or Mann–Whitney *U* test as appropriate. Pictorial presentations of the key results were made using appropriate statistical graphs. All *P* values presented were two-tailed, and *P* values < 0.05 were considered as statistically significant. All Statistical analyses were done using statistical packages SPSS 22.0 (SPSS Inc., Chicago, IL) and Epi-info (Centers for Disease Control and Prevention, Atlanta, GA) software.

## 3. Results

The sample had a mean age of 38.31 ± 10.65 years and was mainly comprised of females, with a female to male ratio of 9.5 to 1.  Table 1 summarizes other demographic and medical data.

Four oral manifestations were observed in more than half of the study sample: gingivitis, periodontal disease, cavitation, and missing teeth. A summary of other oral manifestations is listed in Table 2.

**Figure 1:**
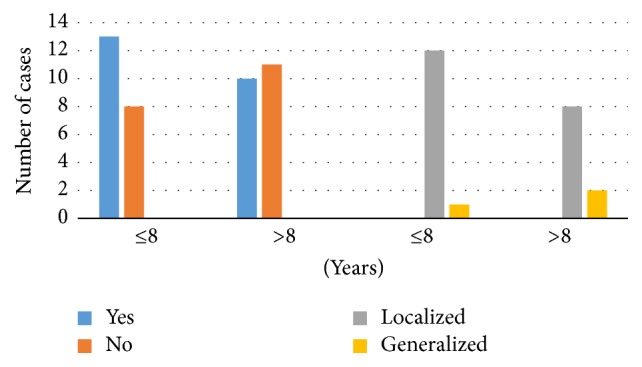
Number of cases and type of gingivitis.

The dental examination revealed that 54.8% (95% CI 39.9, 68.8) of the study sample had a form of gingivitis, of which more of those with an SLE duration of ≤8 years (62% versus 48%) had gingivitis (*P* = 0.352). Localized gingivitis was found more commonly among those with an SLE duration of ≤8 years (57% versus 38%); however this difference was noted to be statistically insignificant (*P* = 0.448).  Figure 1 displays the number of cases with gingivitis as well as the type.

**Figure 2:**
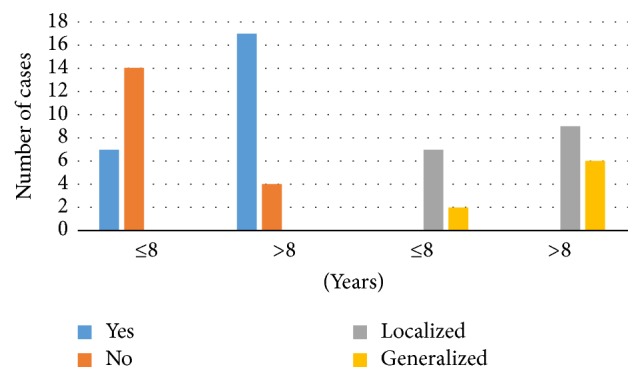
Number of cases and type of periodontal disease.

Furthermore, periodontal disease was observed in 57.1% of the study sample. The presence of periodontal disease and type was compared between those with an SLE duration of ≤8 years and those >8 years as shown in Figure 2. Significantly higher participants with an SLE duration > 8 years had periodontal disease compared to those with SLE duration ≤8 years (81% versus 33%; *P* = 0.002). Localized periodontal disease was found more commonly among those with an SLE duration of > 8 years (43% versus 33%, *P* = 0.119).

**Figure 3:**
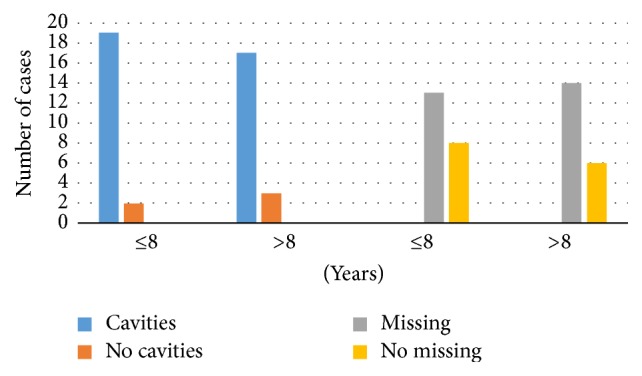
Number of cases with cavities and missing teeth.

Finally, 88.1% of the sample had cavities, while 64.3% had missing teeth.  Figure 3 compares those with an SLE duration ≤ 8 years and those with >8 years, in regard to cavities (90.5% versus 85.7%, *P* = 0.634) and missing teeth (61.9% versus 66.7%, *P* = 0.747).

## 4. Discussion

The current study investigated the presence of oral manifestations among SLE patients in Qatar. The mean age of our study sample was 38.3 ± 10.6 years, and the mean disease duration was 9.4 ± 6.6 years. A study conducted in Saudi Arabia on 624 SLE patients reported a mean age of 34.3 ± 11.9 years, and a mean disease duration of 9.3 ± 5.3 years [26]. The LUMINA cohort reported a mean age of 37.3 ± 12.8 years, and a mean disease duration of 20 ± 17 months [27].

The female to male ratio in our study was 9.5 : 1. This is similar to the ratio reported in China 9.6 : 1 [28], in Saudi Arabia 9.8 : 1 [26], or in the Euro-Lupus cohort 10 : 1 [5]. Lower ratios were reported in Spain 8 : 1 [29] and in Iran 6.5 : 1 [30].

The results of our study showed varying prevalence rates for the different oral manifestations, ranging from 2.4% for soft palate ulcers, cheilitis, and oral candida to 88.1% for the presence of cavities. Soft palate ulcers are one of the criteria for diagnosing SLE and are usually found more commonly in patients with active disease. A study in Iran reported that 54% of the 188 SLE patients had oral mucosal lesions, with the ulcer being the most prevalent (28%) [31]. More than 80% of the 16 SLE patients in another study were reported to have oral manifestations [15]. We had a low rate of oral ulcers in our study, most likely because only 8/42 of the patients were having active disease during the examination.

The results of this study also showed a prevalence rate of 57.1% for periodontal disease (localized 38.1%, generalized 19%). A study conducted in Saudi Arabia reported that there was no difference in periodontal parameters between 25 SLE patients and 50 healthy controls [25]. Another Saudi study reported a 68% prevalence rate of periodontitis, among a sample of 282 of Saudi dental school patients. The localized form of periodontitis was present in 28% of the sample, while the generalized form was present in 40% [32]. A study conducted in Japan reported a 70% prevalence rate of periodontitis among SLE patients compared to 30% among the general population [33]. An older case report involving a patient with active SLE reported that the patient suffered from severe and generalized gingival recession and periodontal involvement [34]. An older study showed that 93.8% of SLE patients had periodontitis [15].

Furthermore, 64.3% of the sample of this study had missing teeth. A study conducted in Brazil recently showed a higher rate of missing teeth among 75 SLE patients compared to 78 participants without SLE. However, no differences were found between the two groups in regard to the presence of periodontitis, decayed and/or filled teeth in the same study [35]. Moreover, 88.1% of our study sample had cavities, which comes concurrent with a recent study conducted in Mexico which reported that 85% of the 60 SLE patients had cavities, with the rate reaching 100% among those with active disease [36].

Among the challenges that this study faced was the scarcity of SLE patients. In addition, due to the nature of the disease, majorly being dominant by females, a very low number of male patients were recruited for this study, which did not allow for comparisons based on gender. Among the disadvantages, due to the small sample size, it would be difficult to generalize any of the results obtained in this study to other SLE patients in Qatar or in the region.

Future studies will need to be multicenter studies in order to secure a larger sample size. Additionally, we were not able to compare to a control group, which would be a good idea to include in future studies. Finally, longitudinal studies are needed in order to ascertain any causation factors or common etiology pathways.

In regard to recommendations, researchers recommend examining mucosal changes, periodontal inflammation and bleeding, and overall dentition of systemic diseases such as SLE, as it would allow for early detection and treatment [14, 31]. Healthcare providers are also recommended to monitor infections of the head and neck to avoid further progression of any possible infection due to the higher predisposition to complications because of immunosuppressive treatment received [10].

## 5. Conclusion

This pilot study found high rates of gingivitis, periodontal disease, cavities, and missing teeth among SLE patients in Qatar. It is recommended that healthcare providers of such patients monitor the presence of any oral manifestations in order to arrange for early treatment. Future longitudinal studies are needed in order to ascertain any causation factors or common etiology pathways.

## Figures and Tables

**Table 1:** Demographic and medical data of the study sample.

	*N*	%
*Age (years)*		
18–29	11	26.2
30–49	25	59.5
≥50	6	14.3

*Gender*		
Male	4	9.5
Female	38	90.5

*Education*		
High school	16	38.1
University	26	61.9

*SLE duration*		
≤8 years	21	50.0
>8 years	21	50.0

*SLE activity*		
Active	8	19.0
Inactive	34	81.0

*On steroids*		
No	18	42.9
Yes		
1–10 mg	20	47.6
11–30 mg	3	7.1
≥31	1	2.4

*On immunosuppressant*		
Yes	15	35.7
No	27	64.3

*Dry mouth*		
Yes	14	33.3
No	28	46.7

**Table 2:** A summary of oral manifestations of the study sample.

	*N*	% (95% CI)
*Hard palate ulcer*		
Yes	2	4.8 (1.3, 15.8)
No	40	95.2 (84.2, 98.7)

*Soft palate ulcer*		
Yes	1	2.4 (0.43, 12.59)
No	41	97.6 (87.7, 99.6)

*Ecchymosis*		
Yes	0	0 (0, 8.38)
No	42	100.0 (91.6, 100)

*Petechiae*		
Yes	7	16.7 (8.32, 30.6)
No	35	83.3 (69.4, 91.7)

*Lupus mucosae oris*		
Yes	0	0 (0, 8.38)
No	42	100.0 (91.6, 100)

*Cheilitis*		
Yes	1	2.4 (0.43, 12.59)
No	41	97.6 (87.7, 99.6)

*Candida*		
Yes	1	2.4 (0.43, 12.59)
No	41	97.6 (87.7, 99.6)

*Discoid*		
Yes	0	0 (0, 8.38)
No	42	100.0 (91.6, 100)

*Herpes simplex*		
Yes	0	0 (0, 8.38)
No	42	100.0 (91.6, 100)

*Gingivitis*		
Yes	23	54.8 (39.9, 68.8)
No	19	45.2 (31.2, 60.1)

*Gingivitis type*		
Localized	20	47.6 (33.4, 62.3)
Generalized	3	7.1 (2.5, 19.1)

*Periodontal disease*		
Yes	24	57.1 (42.2, 70.9)
No	18	42.9 (29.1, 57.8)

*Periodontal disease type*		
Localized	16	38.1 (25.0, 53.2)
Generalized	8	19.0 (9.9, 33.3)

*Cavities*		
Yes	37	88.1 (75.0, 94.8)
No	5	11.9 (5.2, 25.0)

*Missing teeth*		
Yes	27	64.3 (49.2, 77.0)
No	15	35.7 (22.9, 50.8)

CI: confidence interval.

## Abbreviations

ACR: American College of Rheumatology
PD: Periodontal disease
SLE: Systemic lupus erythematosus.
